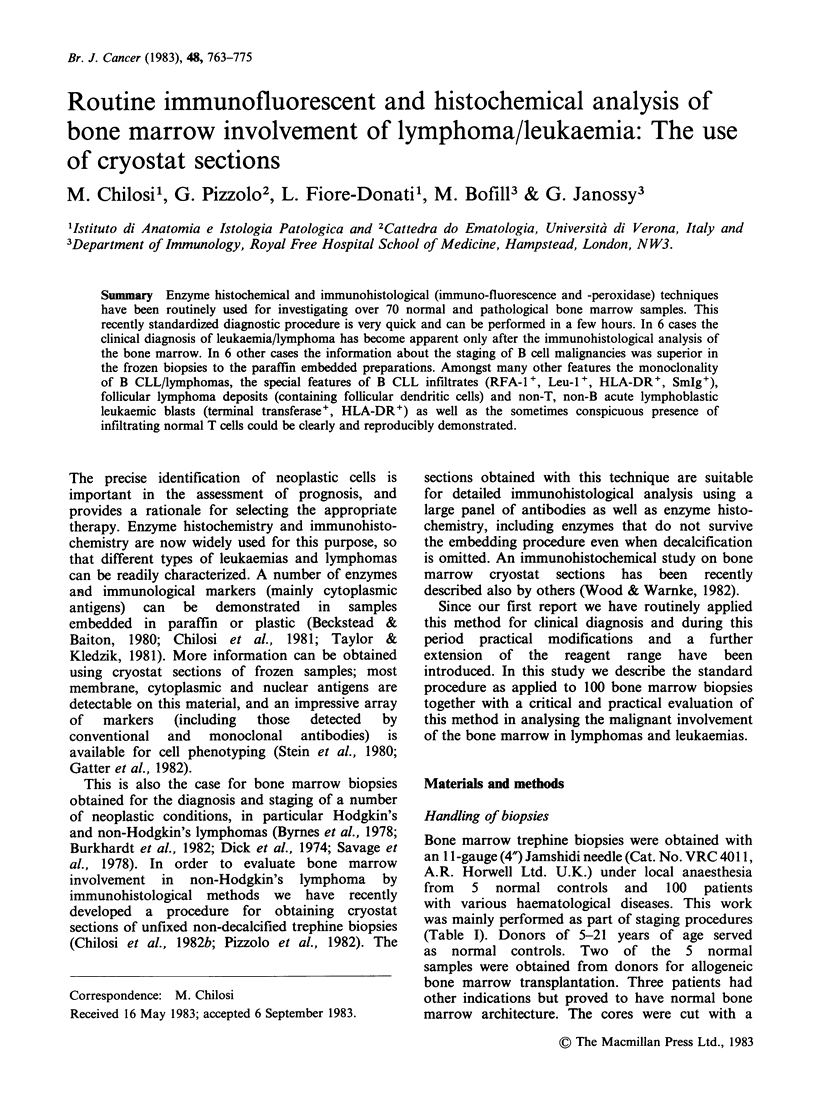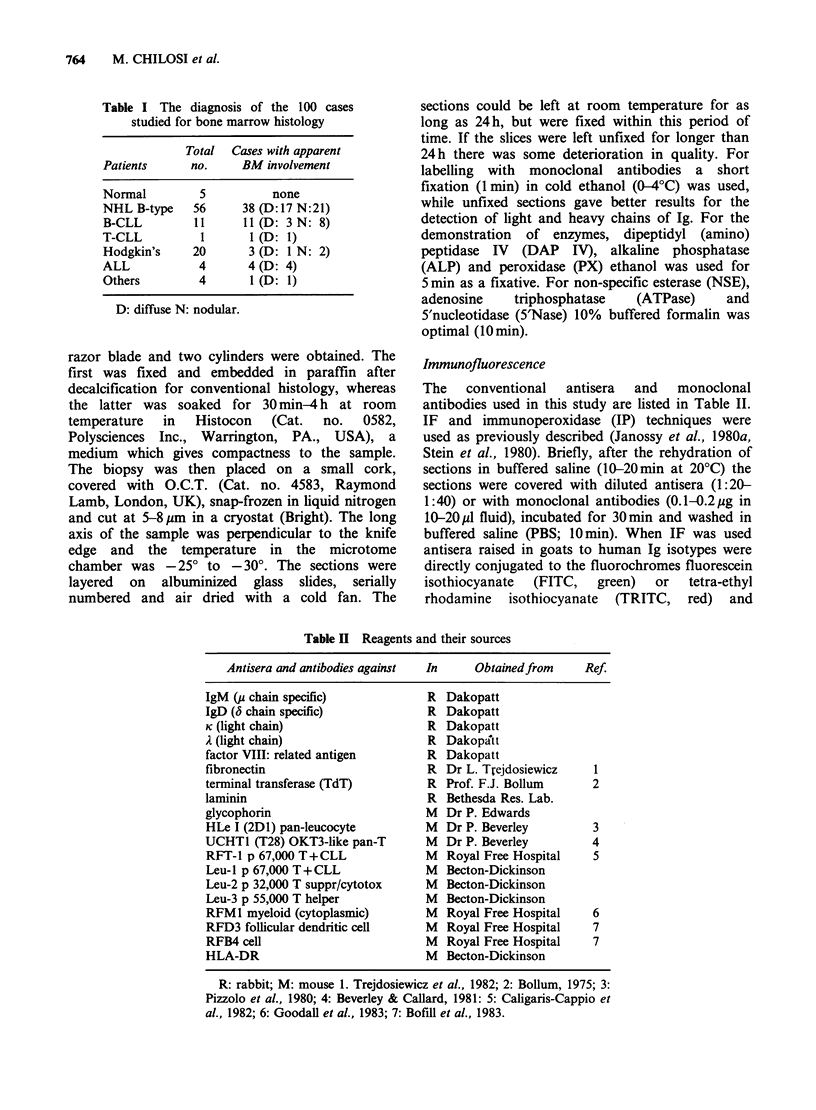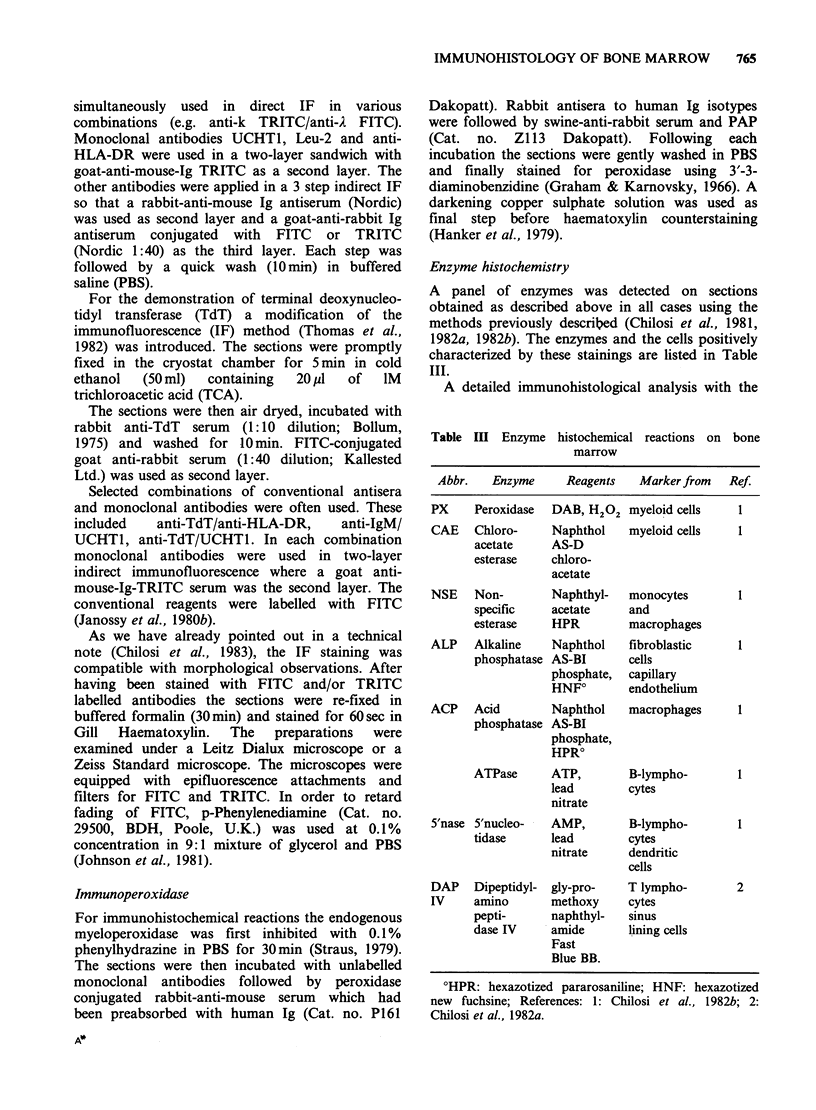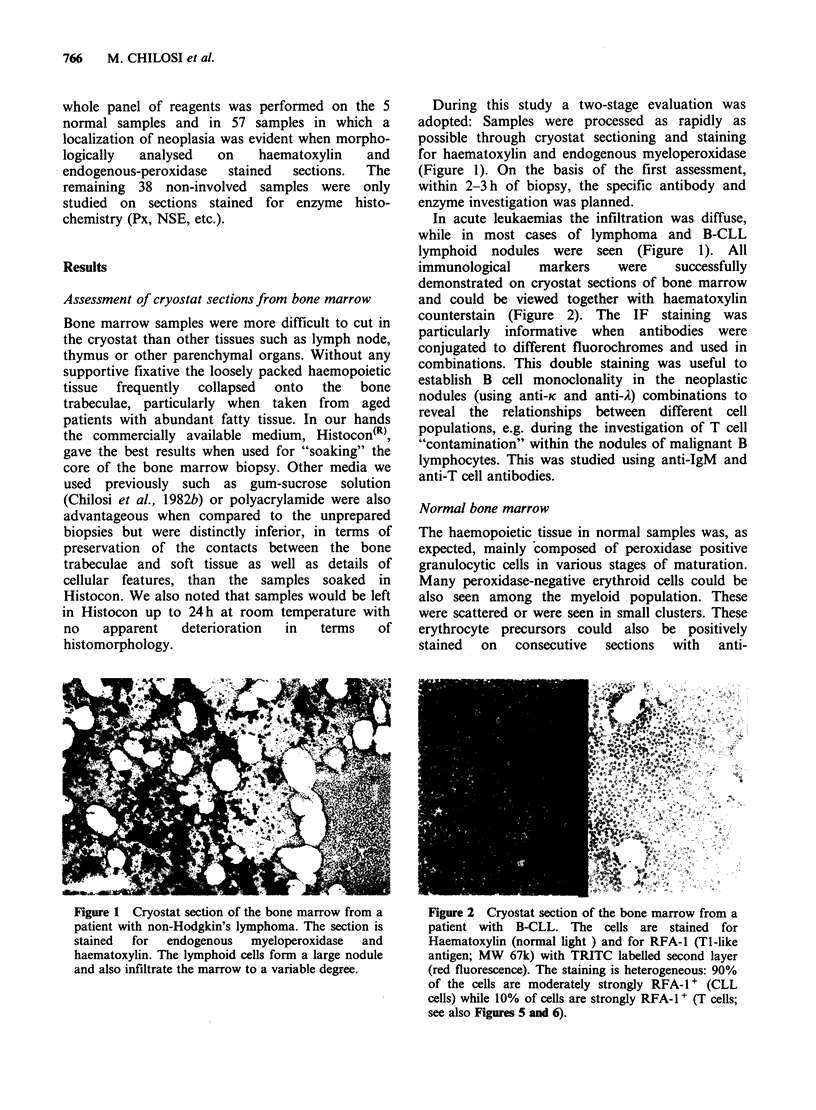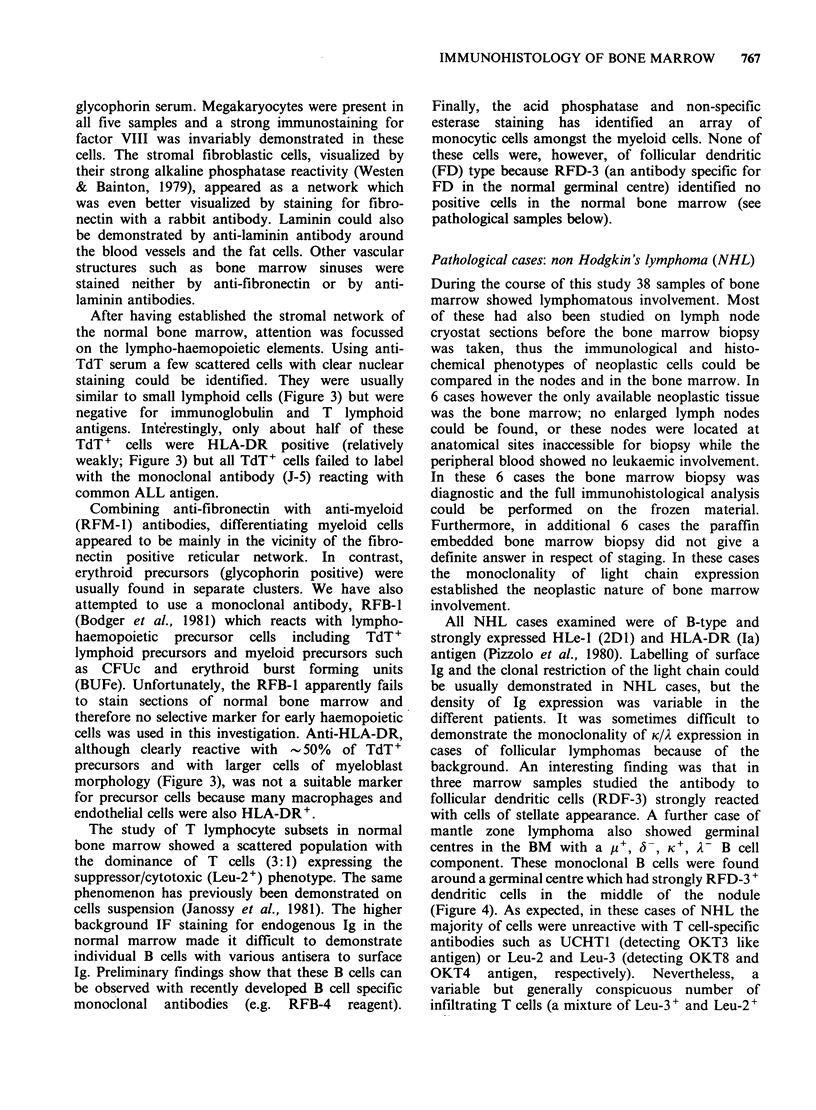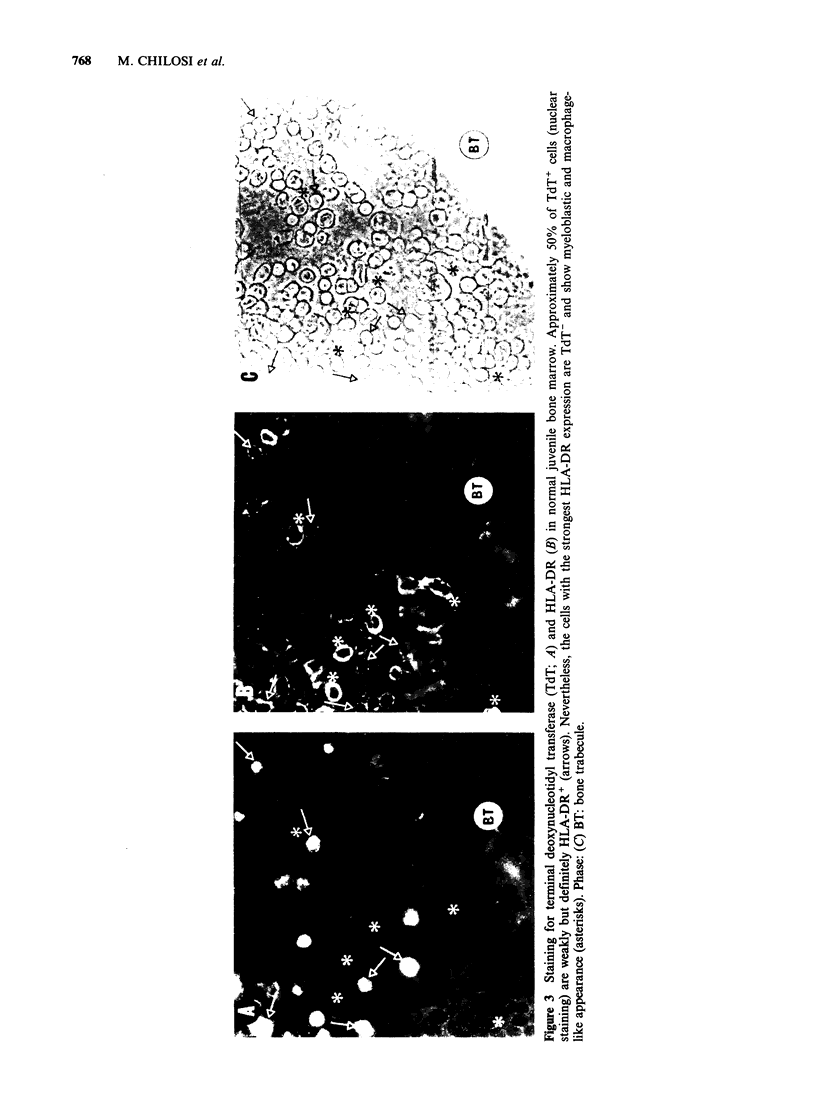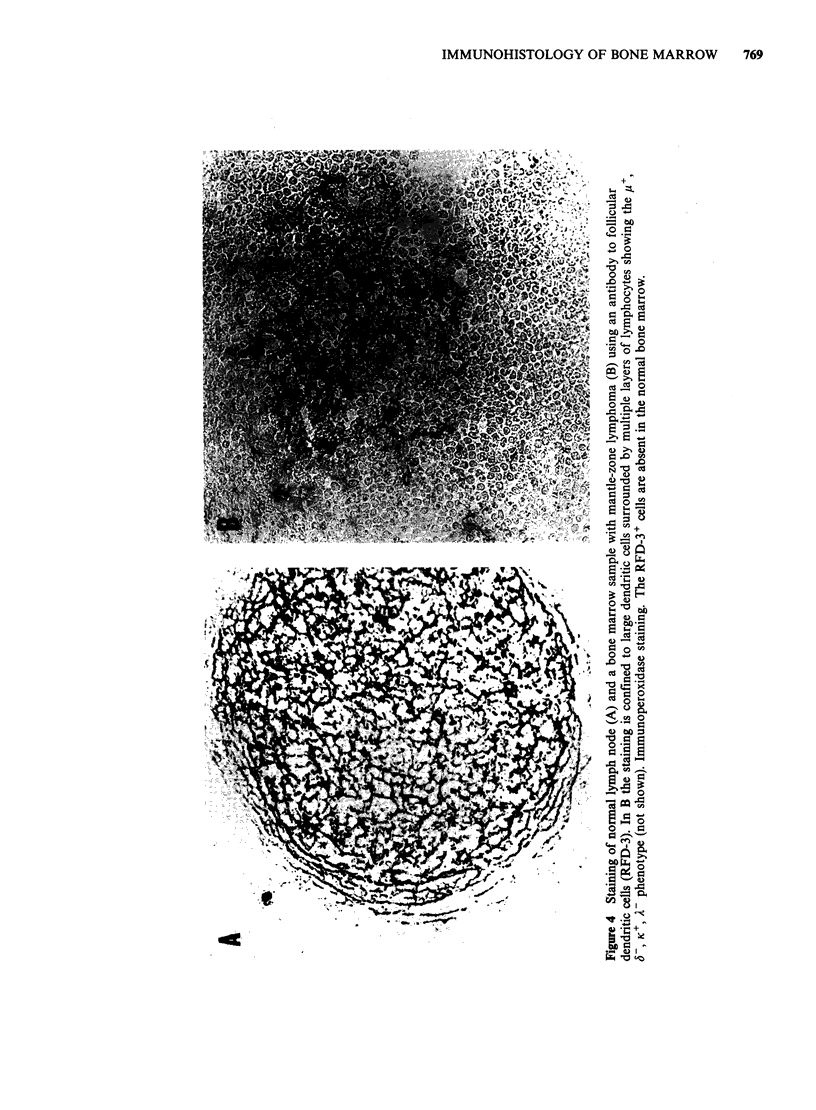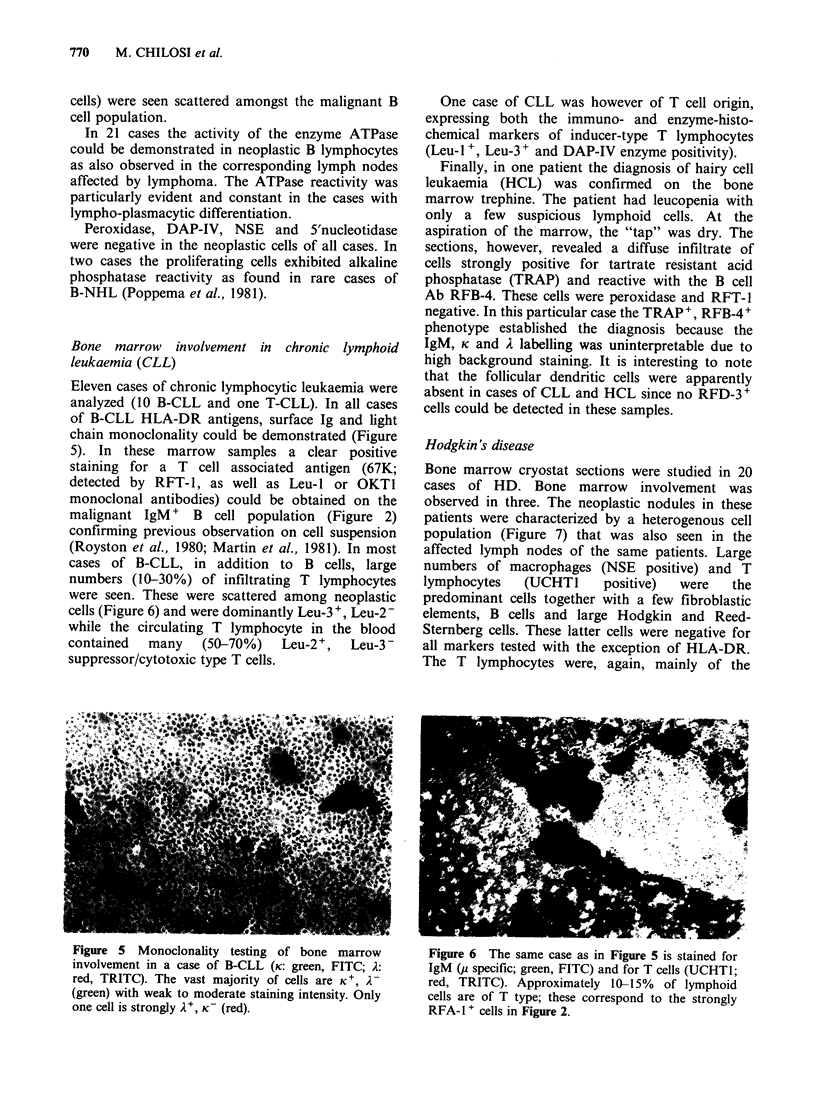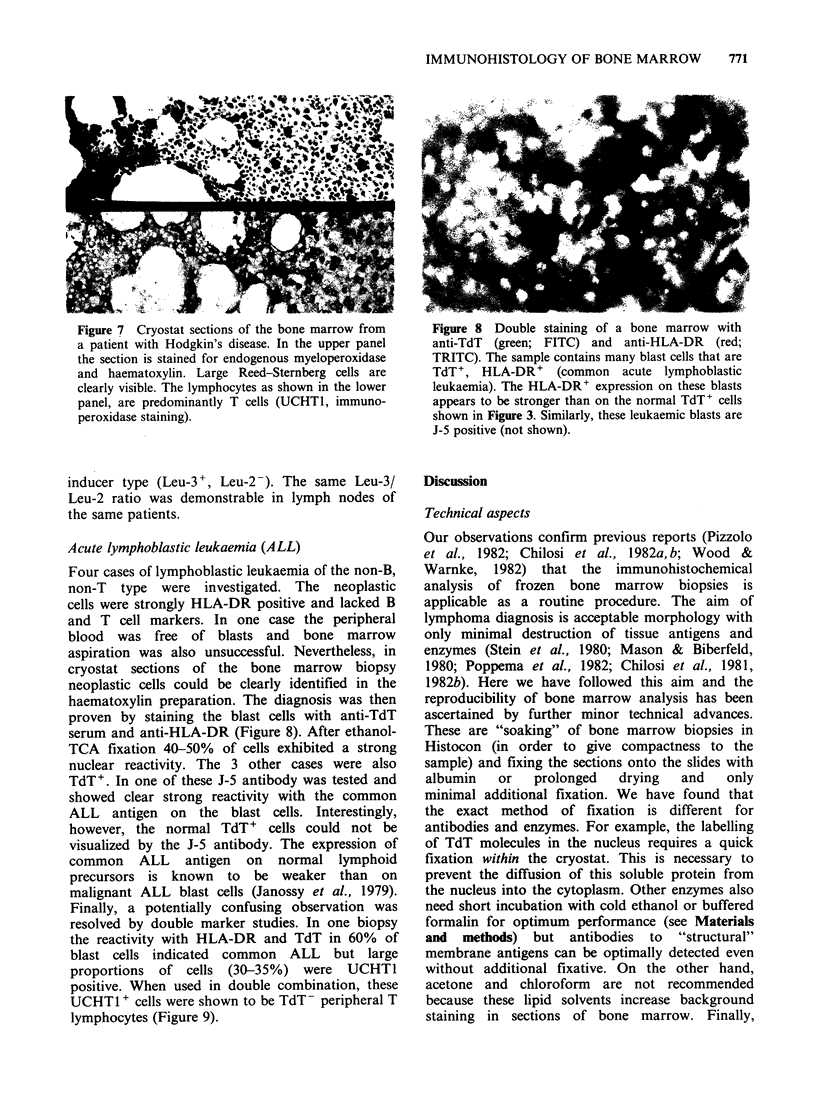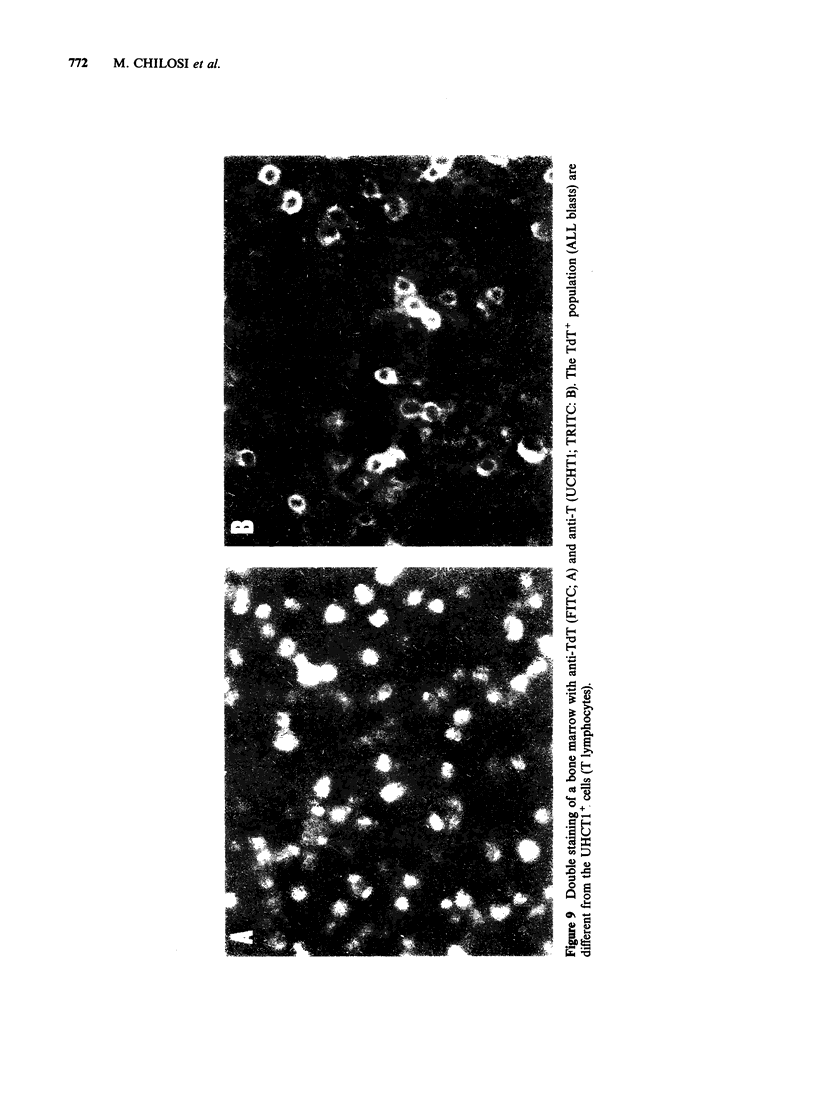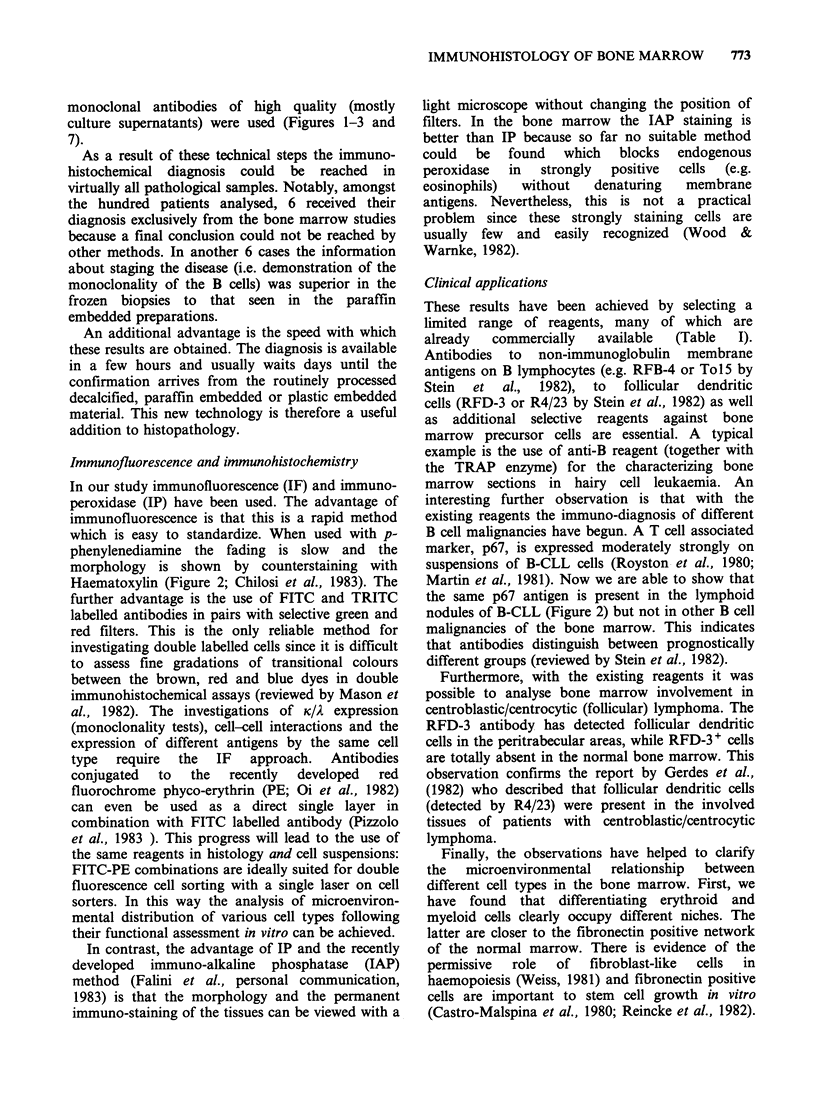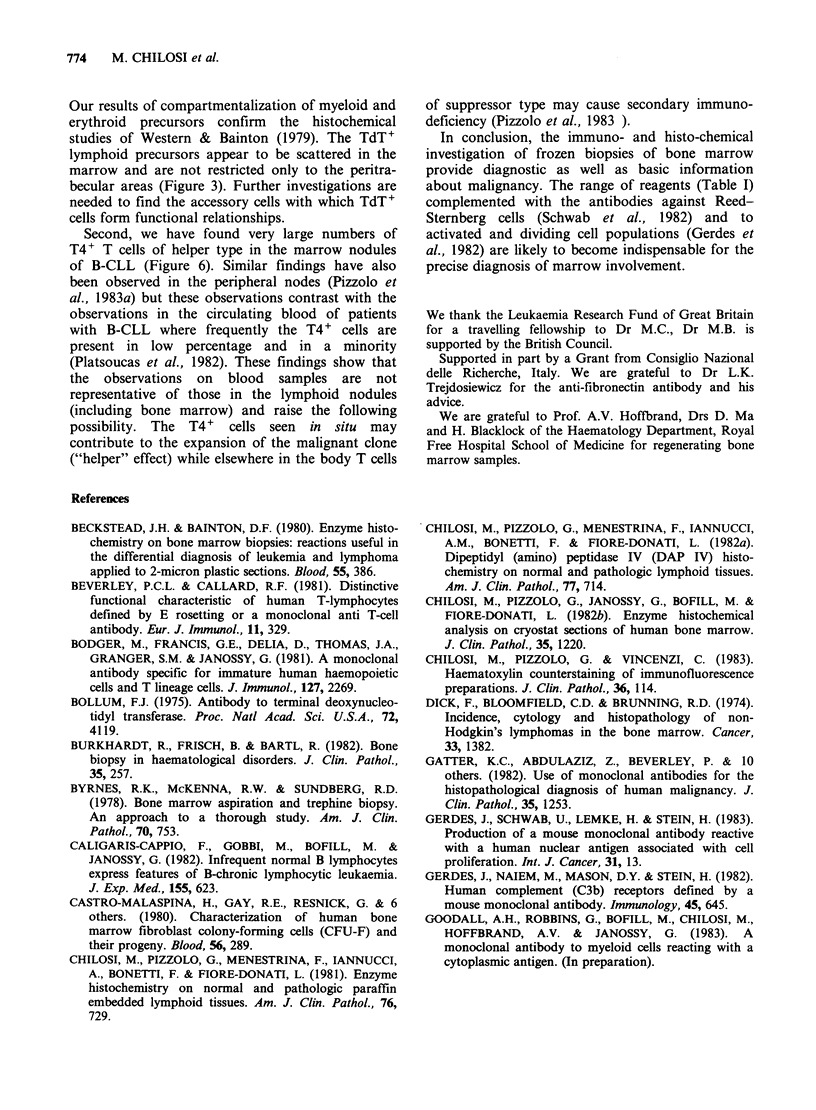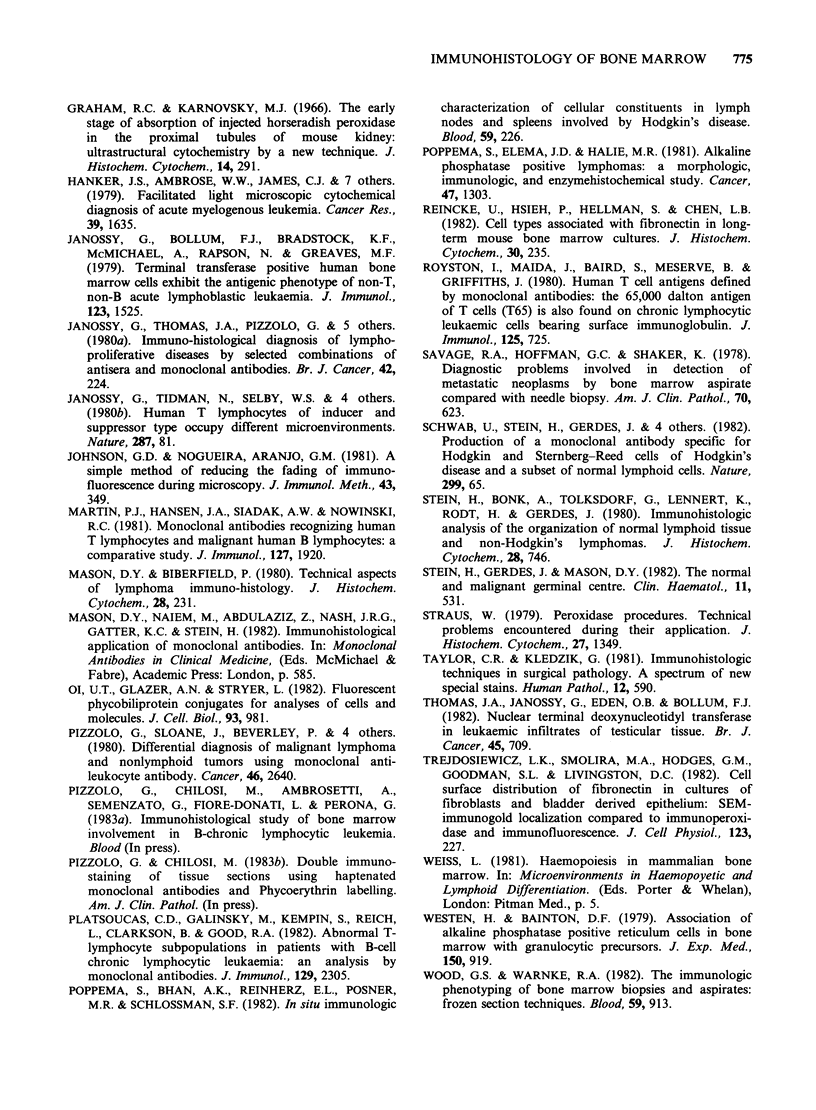# Routine immunofluorescent and histochemical analysis of bone marrow involvement of lymphoma/leukaemia: the use of cryostat sections.

**DOI:** 10.1038/bjc.1983.265

**Published:** 1983-12

**Authors:** M. Chilosi, G. Pizzolo, L. Fiore-Donati, M. Bofill, G. Janossy

## Abstract

**Images:**


*Br. J. Cancer* (1983), **48**, 763–775

### Routine immunofluorescent and histochemical analysis of bone marrow involvement of lymphoma/leukaemia: The use of cryostat sections

M. Chilosi[1], G. Pizzolo[2], L. Fiore-Donati[1], M. Bofill[3] & G. Janossy[3]

[1]*Istituto di Anatomia e Istologia Patologica and* [2]*Cattedra do Ematologia, Università di Verona, Italy and* [3]*Department of Immunology, Royal Free Hospital School of Medicine, Hampstead, London, NW3.*

**Summary** Enzyme histochemical and immunohistological (immuno-fluorescence and -peroxidase) techniques have been routinely used for investigating over 70 normal and pathological bone marrow samples. This recently standardized diagnostic procedure is very quick and can be performed in a few hours. In 6 cases the clinical diagnosis of leukaemia/lymphoma has become apparent only after the immunohistological analysis of the bone marrow. In 6 other cases the information about the staging of B cell malignancies was superior in the frozen biopsies to the paraffin embedded preparations. Amongst many other features the monoclonality of B CLL/lymphomas, the special features of B CLL infiltrates (RFA-1$^+$, Leu-1$^+$, HLA-DR$^+$, SmIg$^+$), follicular lymphoma deposits (containing follicular dendritic cells) and non-T, non-B acute lymphoblastic leukaemic blasts (terminal transferase$^+$, HLA-DR$^+$) as well as the sometimes conspicuous presence of infiltrating normal T cells could be clearly and reproducibly demonstrated.

The precise identification of neoplastic cells is important in the assessment of prognosis, and provides a rationale for selecting the appropriate therapy. Enzyme histochemistry and immunohistochemistry are now widely used for this purpose, so that different types of leukaemias and lymphomas can be readily characterized. A number of enzymes and immunological markers (mainly cytoplasmic antigens) can be demonstrated in samples embedded in paraffin or plastic (Beckstead & Baiton, 1980; Chilosi *et al.*, 1981; Taylor & Kledzik, 1981). More information can be obtained using cryostat sections of frozen samples; most membrane, cytoplasmic and nuclear antigens are detectable on this material, and an impressive array of markers (including those detected by conventional and monoclonal antibodies) is available for cell phenotyping (Stein *et al.*, 1980; Gatter *et al.*, 1982).

This is also the case for bone marrow biopsies obtained for the diagnosis and staging of a number of neoplastic conditions, in particular Hodgkin's and non-Hodgkin's lymphomas (Byrnes *et al.*, 1978; Burkhardt *et al.*, 1982; Dick *et al.*, 1974; Savage *et al.*, 1978). In order to evaluate bone marrow involvement in non-Hodgkin's lymphoma by immunohistological methods we have recently developed a procedure for obtaining cryostat sections of unfixed non-decalcified trephine biopsies (Chilosi *et al.*, 1982*b*; Pizzolo *et al.*, 1982). The sections obtained with this technique are suitable for detailed immunohistological analysis using a large panel of antibodies as well as enzyme histochemistry, including enzymes that do not survive the embedding procedure even when decalcification is omitted. An immunohistochemical study on bone marrow cryostat sections has been recently described also by others (Wood & Warnke, 1982).

Since our first report we have routinely applied this method for clinical diagnosis and during this period practical modifications and a further extension of the reagent range have been introduced. In this study we describe the standard procedure as applied to 100 bone marrow biopsies together with a critical and practical evaluation of this method in analysing the malignant involvement of the bone marrow in lymphomas and leukaemias.

### Materials and methods

### *Handling of biopsies*

Bone marrow trephine biopsies were obtained with an 11-gauge (4″) Jamshidi needle (Cat. No. VRC 4011, A.R. Horwell Ltd. U.K.) under local anaesthesia from 5 normal controls and 100 patients with various haematological diseases. This work was mainly performed as part of staging procedures (Table I). Donors of 5–21 years of age served as normal controls. Two of the 5 normal samples were obtained from donors for allogeneic bone marrow transplantation. Three patients had other indications but proved to have normal bone marrow architecture. The cores were cut with a

Correspondence: M. Chilosi
Received 16 May 1983; accepted 6 September 1983.

© The Macmillan Press Ltd., 1983

**Table I** The diagnosis of the 100 cases studied for bone marrow histology

| *Patients* | *Total no.* | *Cases with apparent BM involvement* |
|---|---|---|
| Normal | 5 | none |
| NHL B-type | 56 | 38 (D:17 N:21) |
| B-CLL | 11 | 11 (D: 3 N: 8) |
| T-CLL | 1 | 1 (D: 1) |
| Hodgkin's | 20 | 3 (D: 1 N: 2) |
| ALL | 4 | 4 (D: 4) |
| Others | 4 | 1 (D: 1) |

D: diffuse N: nodular.

razor blade and two cylinders were obtained. The first was fixed and embedded in paraffin after decalcification for conventional histology, whereas the latter was soaked for 30 min–4 h at room temperature in Histocon (Cat. no. 0582, Polysciences Inc., Warrington, PA., USA), a medium which gives compactness to the sample. The biopsy was then placed on a small cork, covered with O.C.T. (Cat. no. 4583, Raymond Lamb, London, UK), snap-frozen in liquid nitrogen and cut at 5–8 μm in a cryostat (Bright). The long axis of the sample was perpendicular to the knife edge and the temperature in the microtome chamber was −25° to −30°. The sections were layered on albuminized glass slides, serially numbered and air dried with a cold fan. The sections could be left at room temperature for as long as 24 h, but were fixed within this period of time. If the slices were left unfixed for longer than 24 h there was some deterioration in quality. For labelling with monoclonal antibodies a short fixation (1 min) in cold ethanol (0–4°C) was used, while unfixed sections gave better results for the detection of light and heavy chains of Ig. For the demonstration of enzymes, dipeptidyl (amino) peptidase IV (DAP IV), alkaline phosphatase (ALP) and peroxidase (PX) ethanol was used for 5 min as a fixative. For non-specific esterase (NSE), adenosine triphosphatase (ATPase) and 5′nucleotidase (5′Nase) 10% buffered formalin was optimal (10 min).

*Immunofluorescence*

The conventional antisera and monoclonal antibodies used in this study are listed in Table II. IF and immunoperoxidase (IP) techniques were used as previously described (Janossy *et al.*, 1980*a*, Stein *et al.*, 1980). Briefly, after the rehydration of sections in buffered saline (10–20 min at 20°C) the sections were covered with diluted antisera (1:20–1:40) or with monoclonal antibodies (0.1–0.2 μg in 10–20 μl fluid), incubated for 30 min and washed in buffered saline (PBS; 10 min). When IF was used antisera raised in goats to human Ig isotypes were directly conjugated to the fluorochromes fluorescein isothiocyanate (FITC, green) or tetra-ethyl rhodamine isothiocyanate (TRITC, red) and

**Table II** Reagents and their sources

| *Antisera and antibodies against* | *In* | *Obtained from* | *Ref.* |
|---|---|---|---|
| IgM (μ chain specific) | R | Dakopatt | |
| IgD (δ chain specific) | R | Dakopatt | |
| κ (light chain) | R | Dakopatt | |
| λ (light chain) | R | Dakopatt | |
| factor VIII: related antigen | R | Dakopatt | |
| fibronectin | R | Dr L. Trejdosiewicz | 1 |
| terminal transferase (TdT) | R | Prof. F.J. Bollum | 2 |
| laminin | R | Bethesda Res. Lab. | |
| glycophorin | M | Dr P. Edwards | |
| HLe I (2D1) pan-leucocyte | M | Dr P. Beverley | 3 |
| UCHT1 (T28) OKT3-like pan-T | M | Dr P. Beverley | 4 |
| RFT-1 p 67,000 T+CLL | M | Royal Free Hospital | 5 |
| Leu-1 p 67,000 T+CLL | M | Becton-Dickinson | |
| Leu-2 p 32,000 T suppr/cytotox | M | Becton-Dickinson | |
| Leu-3 p 55,000 T helper | M | Becton-Dickinson | |
| RFM1 myeloid (cytoplasmic) | M | Royal Free Hospital | 6 |
| RFD3 follicular dendritic cell | M | Royal Free Hospital | 7 |
| RFB4 cell | M | Royal Free Hospital | 7 |
| HLA-DR | M | Becton-Dickinson | |

R: rabbit; M: mouse 1. Trejdosiewicz *et al.*, 1982; 2: Bollum, 1975; 3: Pizzolo *et al.*, 1980; 4: Beverley & Callard, 1981: 5: Caligaris-Cappio *et al.*, 1982; 6: Goodall *et al.*, 1983; 7: Bofill *et al.*, 1983.

simultaneously used in direct IF in various combinations (e.g. anti-k TRITC/anti-$\lambda$ FITC). Monoclonal antibodies UCHT1, Leu-2 and anti-HLA-DR were used in a two-layer sandwich with goat-anti-mouse-Ig TRITC as a second layer. The other antibodies were applied in a 3 step indirect IF so that a rabbit-anti-mouse Ig antiserum (Nordic) was used as second layer and a goat-anti-rabbit Ig antiserum conjugated with FITC or TRITC (Nordic 1:40) as the third layer. Each step was followed by a quick wash (10 min) in buffered saline (PBS).

For the demonstration of terminal deoxynucleotidyl transferase (TdT) a modification of the immunofluorescence (IF) method (Thomas *et al.*, 1982) was introduced. The sections were promptly fixed in the cryostat chamber for 5 min in cold ethanol (50 ml) containing 20 $\mu$l of 1M trichloroacetic acid (TCA).

The sections were then air dryed, incubated with rabbit anti-TdT serum (1:10 dilution; Bollum, 1975) and washed for 10 min. FITC-conjugated goat anti-rabbit serum (1:40 dilution; Kallested Ltd.) was used as second layer.

Selected combinations of conventional antisera and monoclonal antibodies were often used. These included anti-TdT/anti-HLA-DR, anti-IgM/UCHT1, anti-TdT/UCHT1. In each combination monoclonal antibodies were used in two-layer indirect immunofluorescence where a goat anti-mouse-Ig-TRITC serum was the second layer. The conventional reagents were labelled with FITC (Janossy *et al.*, 1980*b*).

As we have already pointed out in a technical note (Chilosi *et al.*, 1983), the IF staining was compatible with morphological observations. After having been stained with FITC and/or TRITC labelled antibodies the sections were re-fixed in buffered formalin (30 min) and stained for 60 sec in Gill Haematoxylin. The preparations were examined under a Leitz Dialux microscope or a Zeiss Standard microscope. The microscopes were equipped with epifluorescence attachments and filters for FITC and TRITC. In order to retard fading of FITC, p-Phenylenediamine (Cat. no. 29500, BDH, Poole, U.K.) was used at 0.1% concentration in 9:1 mixture of glycerol and PBS (Johnson *et al.*, 1981).

### *Immunoperoxidase*

For immunohistochemical reactions the endogenous myeloperoxidase was first inhibited with 0.1% phenylhydrazine in PBS for 30 min (Straus, 1979). The sections were then incubated with unlabelled monoclonal antibodies followed by peroxidase conjugated rabbit-anti-mouse serum which had been preabsorbed with human Ig (Cat. no. P161 Dakopatt). Rabbit antisera to human Ig isotypes were followed by swine-anti-rabbit serum and PAP (Cat. no. Z113 Dakopatt). Following each incubation the sections were gently washed in PBS and finally stained for peroxidase using 3′-3-diaminobenzidine (Graham & Karnovsky, 1966). A darkening copper sulphate solution was used as final step before haematoxylin counterstaining (Hanker *et al.*, 1979).

### *Enzyme histochemistry*

A panel of enzymes was detected on sections obtained as described above in all cases using the methods previously described (Chilosi *et al.*, 1981, 1982*a*, 1982*b*). The enzymes and the cells positively characterized by these stainings are listed in Table III.

A detailed immunohistological analysis with the

**Table III** Enzyme histochemical reactions on bone marrow

| *Abbr.* | *Enzyme* | *Reagents* | *Marker from* | *Ref.* |
|---|---|---|---|---|
| PX | Peroxidase | DAB, $H_2O_2$ | myeloid cells | 1 |
| CAE | Chloro-acetate esterase | Naphthol AS-D chloro-acetate | myeloid cells | 1 |
| NSE | Non-specific esterase | Naphthyl-acetate HPR | monocytes and macrophages | 1 |
| ALP | Alkaline phosphatase | Naphthol AS-BI phosphate, HNF° | fibroblastic cells capillary endothelium | 1 |
| ACP | Acid phosphatase | Naphthol AS-BI phosphate, HPR° | macrophages | 1 |
| | ATPase | ATP, lead nitrate | B-lymphocytes | 1 |
| 5′nase | 5′nucleotidase | AMP, lead nitrate | B-lymphocytes dendritic cells | 1 |
| DAP IV | Dipeptidyl-amino peptidase IV | gly-pro-methoxy naphthyl-amide Fast Blue BB. | T lymphocytes sinus lining cells | 2 |

°HPR: hexazotized pararosaniline; HNF: hexazotized new fuchsine; References: 1: Chilosi *et al.*, 1982*b*; 2: Chilosi *et al.*, 1982*a*.

whole panel of reagents was performed on the 5 normal samples and in 57 samples in which a localization of neoplasia was evident when morphologically analysed on haematoxylin and endogenous-peroxidase stained sections. The remaining 38 non-involved samples were only studied on sections stained for enzyme histochemistry (Px, NSE, etc.).

### Results

### *Assessment of cryostat sections from bone marrow*

Bone marrow samples were more difficult to cut in the cryostat than other tissues such as lymph node, thymus or other parenchymal organs. Without any supportive fixative the loosely packed haemopoietic tissue frequently collapsed onto the bone trabeculae, particularly when taken from aged patients with abundant fatty tissue. In our hands the commercially available medium, Histocon$^{(R)}$, gave the best results when used for "soaking" the core of the bone marrow biopsy. Other media we used previously such as gum-sucrose solution (Chilosi *et al.,* 1982*b*) or polyacrylamide were also advantageous when compared to the unprepared biopsies but were distinctly inferior, in terms of preservation of the contacts between the bone trabeculae and soft tissue as well as details of cellular features, than the samples soaked in Histocon. We also noted that samples would be left in Histocon up to 24 h at room temperature with no apparent deterioration in terms of histomorphology.

During this study a two-stage evaluation was adopted: Samples were processed as rapidly as possible through cryostat sectioning and staining for haematoxylin and endogenous myeloperoxidase (Figure 1). On the basis of the first assessment, within 2–3 h of biopsy, the specific antibody and enzyme investigation was planned.

In acute leukaemias the infiltration was diffuse, while in most cases of lymphoma and B-CLL lymphoid nodules were seen (Figure 1). All immunological markers were successfully demonstrated on cryostat sections of bone marrow and could be viewed together with haematoxylin counterstain (Figure 2). The IF staining was particularly informative when antibodies were conjugated to different fluorochromes and used in combinations. This double staining was useful to establish B cell monoclonality in the neoplastic nodules (using anti-$\kappa$ and anti-$\lambda$) combinations to reveal the relationships between different cell populations, e.g. during the investigation of T cell "contamination" within the nodules of malignant B lymphocytes. This was studied using anti-IgM and anti-T cell antibodies.

### *Normal bone marrow*

The haemopoietic tissue in normal samples was, as expected, mainly composed of peroxidase positive granulocytic cells in various stages of maturation. Many peroxidase-negative erythroid cells could be also seen among the myeloid population. These were scattered or were seen in small clusters. These erythrocyte precursors could also be positively stained on consecutive sections with anti-

**Figure 1** Cryostat section of the bone marrow from a patient with non-Hodgkin's lymphoma. The section is stained for endogenous myeloperoxidase and haematoxylin. The lymphoid cells form a large nodule and also infiltrate the marrow to a variable degree.

**Figure 2** Cryostat section of the bone marrow from a patient with B-CLL. The cells are stained for Haematoxylin (normal light ) and for RFA-1 (T1-like antigen; MW 67k) with TRITC labelled second layer (red fluorescence). The staining is heterogeneous: 90% of the cells are moderately strongly RFA-1$^+$ (CLL cells) while 10% of cells are strongly RFA-1$^+$ (T cells; see also **Figures 5 and 6**).

glycophorin serum. Megakaryocytes were present in all five samples and a strong immunostaining for factor VIII was invariably demonstrated in these cells. The stromal fibroblastic cells, visualized by their strong alkaline phosphatase reactivity (Westen & Bainton, 1979), appeared as a network which was even better visualized by staining for fibronectin with a rabbit antibody. Laminin could also be demonstrated by anti-laminin antibody around the blood vessels and the fat cells. Other vascular structures such as bone marrow sinuses were stained neither by anti-fibronectin or by anti-laminin antibodies.

After having established the stromal network of the normal bone marrow, attention was focussed on the lympho-haemopoietic elements. Using anti-TdT serum a few scattered cells with clear nuclear staining could be identified. They were usually similar to small lymphoid cells (Figure 3) but were negative for immunoglobulin and T lymphoid antigens. Interestingly, only about half of these $TdT^+$ cells were HLA-DR positive (relatively weakly; Figure 3) but all $TdT^+$ cells failed to label with the monoclonal antibody (J-5) reacting with common ALL antigen.

Combining anti-fibronectin with anti-myeloid (RFM-1) antibodies, differentiating myeloid cells appeared to be mainly in the vicinity of the fibronectin positive reticular network. In contrast, erythroid precursors (glycophorin positive) were usually found in separate clusters. We have also attempted to use a monoclonal antibody, RFB-1 (Bodger *et al.*, 1981) which reacts with lympho-haemopoietic precursor cells including $TdT^+$ lymphoid precursors and myeloid precursors such as CFUc and erythroid burst forming units (BUFe). Unfortunately, the RFB-1 apparently fails to stain sections of normal bone marrow and therefore no selective marker for early haemopoietic cells was used in this investigation. Anti-HLA-DR, although clearly reactive with $\sim 50\%$ of $TdT^+$ precursors and with larger cells of myeloblast morphology (Figure 3), was not a suitable marker for precursor cells because many macrophages and endothelial cells were also $HLA\text{-}DR^+$.

The study of T lymphocyte subsets in normal bone marrow showed a scattered population with the dominance of T cells (3:1) expressing the suppressor/cytotoxic ($Leu\text{-}2^+$) phenotype. The same phenomenon has previously been demonstrated on cells suspension (Janossy *et al.*, 1981). The higher background IF staining for endogenous Ig in the normal marrow made it difficult to demonstrate individual B cells with various antisera to surface Ig. Preliminary findings show that these B cells can be observed with recently developed B cell specific monoclonal antibodies (e.g. RFB-4 reagent). Finally, the acid phosphatase and non-specific esterase staining has identified an array of monocytic cells amongst the myeloid cells. None of these cells were, however, of follicular dendritic (FD) type because RFD-3 (an antibody specific for FD in the normal germinal centre) identified no positive cells in the normal bone marrow (see pathological samples below).

*Pathological cases: non Hodgkin's lymphoma (NHL)*

During the course of this study 38 samples of bone marrow showed lymphomatous involvement. Most of these had also been studied on lymph node cryostat sections before the bone marrow biopsy was taken, thus the immunological and histochemical phenotypes of neoplastic cells could be compared in the nodes and in the bone marrow. In 6 cases however the only available neoplastic tissue was the bone marrow; no enlarged lymph nodes could be found, or these nodes were located at anatomical sites inaccessible for biopsy while the peripheral blood showed no leukaemic involvement. In these 6 cases the bone marrow biopsy was diagnostic and the full immunohistological analysis could be performed on the frozen material. Furthermore, in additional 6 cases the paraffin embedded bone marrow biopsy did not give a definite answer in respect of staging. In these cases the monoclonality of light chain expression established the neoplastic nature of bone marrow involvement.

All NHL cases examined were of B-type and strongly expressed HLe-1 (2D1) and HLA-DR (Ia) antigen (Pizzolo *et al.*, 1980). Labelling of surface Ig and the clonal restriction of the light chain could be usually demonstrated in NHL cases, but the density of Ig expression was variable in the different patients. It was sometimes difficult to demonstrate the monoclonality of $\kappa/\lambda$ expression in cases of follicular lymphomas because of the background. An interesting finding was that in three marrow samples studied the antibody to follicular dendritic cells (RDF-3) strongly reacted with cells of stellate appearance. A further case of mantle zone lymphoma also showed germinal centres in the BM with a $\mu^+$, $\delta^-$, $\kappa^+$, $\lambda^-$ B cell component. These monoclonal B cells were found around a germinal centre which had strongly $RFD\text{-}3^+$ dendritic cells in the middle of the nodule (Figure 4). As expected, in these cases of NHL the majority of cells were unreactive with T cell-specific antibodies such as UCHT1 (detecting OKT3 like antigen) or Leu-2 and Leu-3 (detecting OKT8 and OKT4 antigen, respectively). Nevertheless, a variable but generally conspicuous number of infiltrating T cells (a mixture of $Leu\text{-}3^+$ and $Leu\text{-}2^+$

cells) were seen scattered amongst the malignant B cell population.

In 21 cases the activity of the enzyme ATPase could be demonstrated in neoplastic B lymphocytes as also observed in the corresponding lymph nodes affected by lymphoma. The ATPase reactivity was particularly evident and constant in the cases with lympho-plasmacytic differentiation.

Peroxidase, DAP-IV, NSE and 5′nucleotidase were negative in the neoplastic cells of all cases. In two cases the proliferating cells exhibited alkaline phosphatase reactivity as found in rare cases of B-NHL (Poppema *et al.*, 1981).

### *Bone marrow involvement in chronic lymphoid leukaemia (CLL)*

Eleven cases of chronic lymphocytic leukaemia were analyzed (10 B-CLL and one T-CLL). In all cases of B-CLL HLA-DR antigens, surface Ig and light chain monoclonality could be demonstrated (Figure 5). In these marrow samples a clear positive staining for a T cell associated antigen (67K; detected by RFT-1, as well as Leu-1 or OKT1 monoclonal antibodies) could be obtained on the malignant IgM$^+$ B cell population (Figure 2) confirming previous observation on cell suspension (Royston *et al.*, 1980; Martin *et al.*, 1981). In most cases of B-CLL, in addition to B cells, large numbers (10–30%) of infiltrating T lymphocytes were seen. These were scattered among neoplastic cells (Figure 6) and were dominantly Leu-3$^+$, Leu-2$^-$ while the circulating T lymphocyte in the blood contained many (50–70%) Leu-2$^+$, Leu-3$^-$ suppressor/cytotoxic type T cells.

One case of CLL was however of T cell origin, expressing both the immuno- and enzyme-histochemical markers of inducer-type T lymphocytes (Leu-1$^+$, Leu-3$^+$ and DAP-IV enzyme positivity).

Finally, in one patient the diagnosis of hairy cell leukaemia (HCL) was confirmed on the bone marrow trephine. The patient had leucopenia with only a few suspicious lymphoid cells. At the aspiration of the marrow, the "tap" was dry. The sections, however, revealed a diffuse infiltrate of cells strongly positive for tartrate resistant acid phosphatase (TRAP) and reactive with the B cell Ab RFB-4. These cells were peroxidase and RFT-1 negative. In this particular case the TRAP$^+$, RFB-4$^+$ phenotype established the diagnosis because the IgM, $\kappa$ and $\lambda$ labelling was uninterpretable due to high background staining. It is interesting to note that the follicular dendritic cells were apparently absent in cases of CLL and HCL since no RFD-3$^+$ cells could be detected in these samples.

### *Hodgkin's disease*

Bone marrow cryostat sections were studied in 20 cases of HD. Bone marrow involvement was observed in three. The neoplastic nodules in these patients were characterized by a heterogenous cell population (Figure 7) that was also seen in the affected lymph nodes of the same patients. Large numbers of macrophages (NSE positive) and T lymphocytes (UCHT1 positive) were the predominant cells together with a few fibroblastic elements, B cells and large Hodgkin and Reed-Sternberg cells. These latter cells were negative for all markers tested with the exception of HLA-DR. The T lymphocytes were, again, mainly of the

**Figure 5** Monoclonality testing of bone marrow involvement in a case of B-CLL ($\kappa$: green, FITC; $\lambda$: red, TRITC). The vast majority of cells are $\kappa^+$, $\lambda^-$ (green) with weak to moderate staining intensity. Only one cell is strongly $\lambda^+$, $\kappa^-$ (red).

**Figure 6** The same case as in **Figure 5** is stained for IgM ($\mu$ specific; green, FITC) and for T cells (UCHT1; red, TRITC). Approximately 10–15% of lymphoid cells are of T type; these correspond to the strongly RFA-1$^+$ cells in **Figure 2**.

**Figure 7** Cryostat sections of the bone marrow from a patient with Hodgkin's disease. In the upper panel the section is stained for endogenous myeloperoxidase and haematoxylin. Large Reed–Sternberg cells are clearly visible. The lymphocytes as shown in the lower panel, are predominantly T cells (UCHT1, immunoperoxidase staining).

**Figure 8** Double staining of a bone marrow with anti-TdT (green; FITC) and anti-HLA-DR (red; TRITC). The sample contains many blast cells that are $TdT^+$, $HLA\text{-}DR^+$ (common acute lymphoblastic leukaemia). The $HLA\text{-}DR^+$ expression on these blasts appears to be stronger than on the normal $TdT^+$ cells shown in **Figure 3**. Similarly, these leukaemic blasts are J-5 positive (not shown).

inducer type ($Leu\text{-}3^+$, $Leu\text{-}2^-$). The same Leu-3/Leu-2 ratio was demonstrable in lymph nodes of the same patients.

*Acute lymphoblastic leukaemia* (*ALL*)

Four cases of lymphoblastic leukaemia of the non-B, non-T type were investigated. The neoplastic cells were strongly HLA-DR positive and lacked B and T cell markers. In one case the peripheral blood was free of blasts and bone marrow aspiration was also unsuccessful. Nevertheless, in cryostat sections of the bone marrow biopsy neoplastic cells could be clearly identified in the haematoxylin preparation. The diagnosis was then proven by staining the blast cells with anti-TdT serum and anti-HLA-DR (Figure 8). After ethanol-TCA fixation 40–50% of cells exhibited a strong nuclear reactivity. The 3 other cases were also $TdT^+$. In one of these J-5 antibody was tested and showed clear strong reactivity with the common ALL antigen on the blast cells. Interestingly, however, the normal $TdT^+$ cells could not be visualized by the J-5 antibody. The expression of common ALL antigen on normal lymphoid precursors is known to be weaker than on malignant ALL blast cells (Janossy *et al.*, 1979). Finally, a potentially confusing observation was resolved by double marker studies. In one biopsy the reactivity with HLA-DR and TdT in 60% of blast cells indicated common ALL but large proportions of cells (30–35%) were UCHT1 positive. When used in double combination, these $UCHT1^+$ cells were shown to be $TdT^-$ peripheral T lymphocytes (Figure 9).

### Discussion

*Technical aspects*

Our observations confirm previous reports (Pizzolo *et al.*, 1982; Chilosi *et al.*, 1982*a,b*; Wood & Warnke, 1982) that the immunohistochemical analysis of frozen bone marrow biopsies is applicable as a routine procedure. The aim of lymphoma diagnosis is acceptable morphology with only minimal destruction of tissue antigens and enzymes (Stein *et al.*, 1980; Mason & Biberfeld, 1980; Poppema *et al.*, 1982; Chilosi *et al.*, 1981, 1982*b*). Here we have followed this aim and the reproducibility of bone marrow analysis has been ascertained by further minor technical advances. These are "soaking" of bone marrow biopsies in Histocon (in order to give compactness to the sample) and fixing the sections onto the slides with albumin or prolonged drying and only minimal additional fixation. We have found that the exact method of fixation is different for antibodies and enzymes. For example, the labelling of TdT molecules in the nucleus requires a quick fixation *within* the cryostat. This is necessary to prevent the diffusion of this soluble protein from the nucleus into the cytoplasm. Other enzymes also need short incubation with cold ethanol or buffered formalin for optimum performance (see **Materials and methods**) but antibodies to "structural" membrane antigens can be optimally detected even without additional fixative. On the other hand, acetone and chloroform are not recommended because these lipid solvents increase background staining in sections of bone marrow. Finally,

**Figure 9** Double staining of a bone marrow with anti-TdT (FITC; A) and anti-T (UCHT1; TRITC: B). The $TdT^+$ population (ALL blasts) are different from the $UHCT1^+$ cells (T lymphocytes).

monoclonal antibodies of high quality (mostly culture supernatants) were used (Figures 1–3 and 7).

As a result of these technical steps the immunohistochemical diagnosis could be reached in virtually all pathological samples. Notably, amongst the hundred patients analysed, 6 received their diagnosis exclusively from the bone marrow studies because a final conclusion could not be reached by other methods. In another 6 cases the information about staging the disease (i.e. demonstration of the monoclonality of the B cells) was superior in the frozen biopsies to that seen in the paraffin embedded preparations.

An additional advantage is the speed with which these results are obtained. The diagnosis is available in a few hours and usually waits days until the confirmation arrives from the routinely processed decalcified, paraffin embedded or plastic embedded material. This new technology is therefore a useful addition to histopathology.

*Immunofluorescence and immunohistochemistry*

In our study immunofluorescence (IF) and immunoperoxidase (IP) have been used. The advantage of immunofluorescence is that this is a rapid method which is easy to standardize. When used with *p*-phenylenediamine the fading is slow and the morphology is shown by counterstaining with Haematoxylin (Figure 2; Chilosi *et al.*, 1983). The further advantage is the use of FITC and TRITC labelled antibodies in pairs with selective green and red filters. This is the only reliable method for investigating double labelled cells since it is difficult to assess fine gradations of transitional colours between the brown, red and blue dyes in double immunohistochemical assays (reviewed by Mason *et al.*, 1982). The investigations of $\kappa/\lambda$ expression (monoclonality tests), cell–cell interactions and the expression of different antigens by the same cell type require the IF approach. Antibodies conjugated to the recently developed red fluorochrome phyco-erythrin (PE; Oi *et al.*, 1982) can even be used as a direct single layer in combination with FITC labelled antibody (Pizzolo *et al.*, 1983 ). This progress will lead to the use of the same reagents in histology *and* cell suspensions: FITC-PE combinations are ideally suited for double fluorescence cell sorting with a single laser on cell sorters. In this way the analysis of microenvironmental distribution of various cell types following their functional assessment *in vitro* can be achieved.

In contrast, the advantage of IP and the recently developed immuno-alkaline phosphatase (IAP) method (Falini *et al.*, personal communication, 1983) is that the morphology and the permanent immuno-staining of the tissues can be viewed with a light microscope without changing the position of filters. In the bone marrow the IAP staining is better than IP because so far no suitable method could be found which blocks endogenous peroxidase in strongly positive cells (e.g. eosinophils) without denaturing membrane antigens. Nevertheless, this is not a practical problem since these strongly staining cells are usually few and easily recognized (Wood & Warnke, 1982).

*Clinical applications*

These results have been achieved by selecting a limited range of reagents, many of which are already commercially available (Table I). Antibodies to non-immunoglobulin membrane antigens on B lymphocytes (e.g. RFB-4 or To15 by Stein *et al.*, 1982), to follicular dendritic cells (RFD-3 or R4/23 by Stein *et al.*, 1982) as well as additional selective reagents against bone marrow precursor cells are essential. A typical example is the use of anti-B reagent (together with the TRAP enzyme) for the characterizing bone marrow sections in hairy cell leukaemia. An interesting further observation is that with the existing reagents the immuno-diagnosis of different B cell malignancies have begun. A T cell associated marker, p67, is expressed moderately strongly on suspensions of B-CLL cells (Royston *et al.*, 1980; Martin *et al.*, 1981). Now we are able to show that the same p67 antigen is present in the lymphoid nodules of B-CLL (Figure 2) but not in other B cell malignancies of the bone marrow. This indicates that antibodies distinguish between prognostically different groups (reviewed by Stein *et al.*, 1982).

Furthermore, with the existing reagents it was possible to analyse bone marrow involvement in centroblastic/centrocytic (follicular) lymphoma. The RFD-3 antibody has detected follicular dendritic cells in the peritrabecular areas, while RFD-3$^+$ cells are totally absent in the normal bone marrow. This observation confirms the report by Gerdes *et al.*, (1982) who described that follicular dendritic cells (detected by R4/23) were present in the involved tissues of patients with centroblastic/centrocytic lymphoma.

Finally, the observations have helped to clarify the microenvironmental relationship between different cell types in the bone marrow. First, we have found that differentiating erythroid and myeloid cells clearly occupy different niches. The latter are closer to the fibronectin positive network of the normal marrow. There is evidence of the permissive role of fibroblast-like cells in haemopoiesis (Weiss, 1981) and fibronectin positive cells are important to stem cell growth *in vitro* (Castro-Malspina *et al.*, 1980; Reincke *et al.*, 1982).

Our results of compartmentalization of myeloid and erythroid precursors confirm the histochemical studies of Western & Bainton (1979). The TdT$^+$ lymphoid precursors appear to be scattered in the marrow and are not restricted only to the peritrabecular areas (Figure 3). Further investigations are needed to find the accessory cells with which TdT$^+$ cells form functional relationships.

Second, we have found very large numbers of T4$^+$ T cells of helper type in the marrow nodules of B-CLL (Figure 6). Similar findings have also been observed in the peripheral nodes (Pizzolo *et al.*, 1983*a*) but these observations contrast with the observations in the circulating blood of patients with B-CLL where frequently the T4$^+$ cells are present in low percentage and in a minority (Platsoucas *et al.*, 1982). These findings show that the observations on blood samples are not representative of those in the lymphoid nodules (including bone marrow) and raise the following possibility. The T4$^+$ cells seen *in situ* may contribute to the expansion of the malignant clone ("helper" effect) while elsewhere in the body T cells of suppressor type may cause secondary immunodeficiency (Pizzolo *et al.*, 1983 ).

In conclusion, the immuno- and histo-chemical investigation of frozen biopsies of bone marrow provide diagnostic as well as basic information about malignancy. The range of reagents (Table I) complemented with the antibodies against Reed–Sternberg cells (Schwab *et al.*, 1982) and to activated and dividing cell populations (Gerdes *et al.*, 1982) are likely to become indispensable for the precise diagnosis of marrow involvement.

We thank the Leukaemia Research Fund of Great Britain for a travelling fellowship to Dr M.C., Dr M.B. is supported by the British Council.

Supported in part by a Grant from Consiglio Nazional delle Richerche, Italy. We are grateful to Dr L.K. Trejdosiewicz for the anti-fibronectin antibody and his advice.

We are grateful to Prof. A.V. Hoffbrand, Drs D. Ma and H. Blacklock of the Haematology Department, Royal Free Hospital School of Medicine for regenerating bone marrow samples.

### References

BECKSTEAD, J.H. & BAINTON, D.F. (1980). Enzyme histochemistry on bone marrow biopsies: reactions useful in the differential diagnosis of leukemia and lymphoma applied to 2-micron plastic sections. *Blood,* **55,** 386.

BEVERLEY, P.C.L. & CALLARD, R.F. (1981). Distinctive functional characteristic of human T-lymphocytes defined by E rosetting or a monoclonal anti T-cell antibody. *Eur. J. Immunol.,* **11,** 329.

BODGER, M., FRANCIS, G.E., DELIA, D., THOMAS, J.A., GRANGER, S.M. & JANOSSY, G. (1981). A monoclonal antibody specific for immature human haemopoietic cells and T lineage cells. *J. Immunol.,* **127,** 2269.

BOLLUM, F.J. (1975). Antibody to terminal deoxynucleotidyl transferase. *Proc. Natl Acad. Sci. U.S.A.,* **72,** 4119.

BURKHARDT, R., FRISCH, B. & BARTL, R. (1982). Bone biopsy in haematological disorders. *J. Clin. Pathol.,* **35,** 257.

BYRNES, R.K., McKENNA, R.W. & SUNDBERG, R.D. (1978). Bone marrow aspiration and trephine biopsy. An approach to a thorough study. *Am. J. Clin. Pathol.,* **70,** 753.

CALIGARIS-CAPPIO, F., GOBBI, M., BOFILL, M. & JANOSSY, G. (1982). Infrequent normal B lymphocytes express features of B-chronic lymphocytic leukaemia. *J. Exp. Med.,* **155,** 623.

CASTRO-MALASPINA, H., GAY, R.E., RESNICK, G. & 6 others. (1980). Characterization of human bone marrow fibroblast colony-forming cells (CFU-F) and their progeny. *Blood,* **56,** 289.

CHILOSI, M., PIZZOLO, G., MENESTRINA, F., IANNUCCI, A., BONETTI, F. & FIORE-DONATI, L. (1981). Enzyme histochemistry on normal and pathologic paraffin embedded lymphoid tissues. *Am. J. Clin. Pathol.,* **76,** 729.

CHILOSI, M., PIZZOLO, G., MENESTRINA, F., IANNUCCI, A.M., BONETTI, F. & FIORE-DONATI, L. (1982*a*). Dipeptidyl (amino) peptidase IV (DAP IV) histochemistry on normal and pathologic lymphoid tissues. *Am. J. Clin. Pathol.,* **77,** 714.

CHILOSI, M., PIZZOLO, G., JANOSSY, G., BOFILL, M. & FIORE-DONATI, L. (1982*b*). Enzyme histochemical analysis on cryostat sections of human bone marrow. *J. Clin. Pathol.,* **35,** 1220.

CHILOSI, M., PIZZOLO, G. & VINCENZI, C. (1983). Haematoxylin counterstaining of immunofluorescence preparations. *J. Clin. Pathol.,* **36,** 114.

DICK, F., BLOOMFIELD, C.D. & BRUNNING, R.D. (1974). Incidence, cytology and histopathology of non-Hodgkin's lymphomas in the bone marrow. *Cancer,* **33,** 1382.

GATTER, K.C., ABDULAZIZ, Z., BEVERLEY, P. & 10 others. (1982). Use of monoclonal antibodies for the histopathological diagnosis of human malignancy. *J. Clin. Pathol.,* **35,** 1253.

GERDES, J., SCHWAB, U., LEMKE, H. & STEIN, H. (1983). Production of a mouse monoclonal antibody reactive with a human nuclear antigen associated with cell proliferation. *Int. J. Cancer,* **31,** 13.

GERDES, J., NAIEM, M., MASON, D.Y. & STEIN, H. (1982). Human complement (C3b) receptors defined by a mouse monoclonal antibody. *Immunology,* **45,** 645.

GOODALL, A.H., ROBBINS, G., BOFILL, M., CHILOSI, M., HOFFBRAND, A.V. & JANOSSY, G. (1983). A monoclonal antibody to myeloid cells reacting with a cytoplasmic antigen. (In preparation).

GRAHAM, R.C. & KARNOVSKY, M.J. (1966). The early stage of absorption of injected horseradish peroxidase in the proximal tubules of mouse kidney: ultrastructural cytochemistry by a new technique. *J. Histochem. Cytochem.*, **14**, 291.

HANKER, J.S., AMBROSE, W.W., JAMES, C.J. & 7 others. (1979). Facilitated light microscopic cytochemical diagnosis of acute myelogenous leukemia. *Cancer Res.*, **39**, 1635.

JANOSSY, G., BOLLUM, F.J., BRADSTOCK, K.F., McMICHAEL, A., RAPSON, N. & GREAVES, M.F. (1979). Terminal transferase positive human bone marrow cells exhibit the antigenic phenotype of non-T, non-B acute lymphoblastic leukaemia. *J. Immunol.*, **123**, 1525.

JANOSSY, G., THOMAS, J.A., PIZZOLO, G. & 5 others. (1980*a*). Immuno-histological diagnosis of lymphoproliferative diseases by selected combinations of antisera and monoclonal antibodies. *Br. J. Cancer*, **42**, 224.

JANOSSY, G., TIDMAN, N., SELBY, W.S. & 4 others. (1980*b*). Human T lymphocytes of inducer and suppressor type occupy different microenvironments. *Nature*, **287**, 81.

JOHNSON, G.D. & NOGUEIRA, ARANJO, G.M. (1981). A simple method of reducing the fading of immunofluorescence during microscopy. *J. Immunol. Meth.*, **43**, 349.

MARTIN, P.J., HANSEN, J.A., SIADAK, A.W. & NOWINSKI, R.C. (1981). Monoclonal antibodies recognizing human T lymphocytes and malignant human B lymphocytes: a comparative study. *J. Immunol.*, **127**, 1920.

MASON, D.Y. & BIBERFIELD, P. (1980). Technical aspects of lymphoma immuno-histology. *J. Histochem. Cytochem.*, **28**, 231.

MASON, D.Y., NAIEM, M., ABDULAZIZ, Z., NASH, J.R.G., GATTER, K.C. & STEIN, H. (1982). Immunohistological application of monoclonal antibodies. In: *Monoclonal Antibodies in Clinical Medicine*, (Eds. McMichael & Fabre), Academic Press: London, p. 585.

OI, U.T., GLAZER, A.N. & STRYER, L. (1982). Fluorescent phycobiliprotein conjugates for analyses of cells and molecules. *J. Cell. Biol.*, **93**, 981.

PIZZOLO, G., SLOANE, J., BEVERLEY, P. & 4 others. (1980). Differential diagnosis of malignant lymphoma and nonlymphoid tumors using monoclonal antileukocyte antibody. *Cancer*, **46**, 2640.

PIZZOLO, G., CHILOSI, M., AMBROSETTI, A., SEMENZATO, G., FIORE-DONATI, L. & PERONA, G. (1983*a*). Immunohistological study of bone marrow involvement in B-chronic lymphocytic leukemia. *Blood* (In press).

PIZZOLO, G. & CHILOSI, M. (1983*b*). Double immunostaining of tissue sections using haptenated monoclonal antibodies and Phycoerythrin labelling. *Am. J. Clin. Pathol.* (In press).

PLATSOUCAS, C.D., GALINSKY, M., KEMPIN, S., REICH, L., CLARKSON, B. & GOOD, R.A. (1982). Abnormal T-lymphocyte subpopulations in patients with B-cell chronic lymphocytic leukaemia: an analysis by monoclonal antibodies. *J. Immunol.*, **129**, 2305.

POPPEMA, S., BHAN, A.K., REINHERZ, E.L., POSNER, M.R. & SCHLOSSMAN, S.F. (1982). *In situ* immunologic characterization of cellular constituents in lymph nodes and spleens involved by Hodgkin's disease. *Blood*, **59**, 226.

POPPEMA, S., ELEMA, J.D. & HALIE, M.R. (1981). Alkaline phosphatase positive lymphomas: a morphologic, immunologic, and enzymehistochemical study. *Cancer*, **47**, 1303.

REINCKE, U., HSIEH, P., HELLMAN, S. & CHEN, L.B. (1982). Cell types associated with fibronectin in long-term mouse bone marrow cultures. *J. Histochem. Cytochem.*, **30**, 235.

ROYSTON, I., MAIDA, J., BAIRD, S., MESERVE, B. & GRIFFITHS, J. (1980). Human T cell antigens defined by monoclonal antibodies: the 65,000 dalton antigen of T cells (T65) is also found on chronic lymphocytic leukaemic cells bearing surface immunoglobulin. *J. Immunol.*, **125**, 725.

SAVAGE, R.A., HOFFMAN, G.C. & SHAKER, K. (1978). Diagnostic problems involved in detection of metastatic neoplasms by bone marrow aspirate compared with needle biopsy. *Am. J. Clin. Pathol.*, **70**, 623.

SCHWAB, U., STEIN, H., GERDES, J. & 4 others. (1982). Production of a monoclonal antibody specific for Hodgkin and Sternberg–Reed cells of Hodgkin's disease and a subset of normal lymphoid cells. *Nature*, **299**, 65.

STEIN, H., BONK, A., TOLKSDORF, G., LENNERT, K., RODT, H. & GERDES, J. (1980). Immunohistologic analysis of the organization of normal lymphoid tissue and non-Hodgkin's lymphomas. *J. Histochem. Cytochem.*, **28**, 746.

STEIN, H., GERDES, J. & MASON, D.Y. (1982). The normal and malignant germinal centre. *Clin. Haematol.*, **11**, 531.

STRAUS, W. (1979). Peroxidase procedures. Technical problems encountered during their application. *J. Histochem. Cytochem.*, **27**, 1349.

TAYLOR, C.R. & KLEDZIK, G. (1981). Immunohistologic techniques in surgical pathology. A spectrum of new special stains. *Human Pathol.*, **12**, 590.

THOMAS, J.A., JANOSSY, G., EDEN, O.B. & BOLLUM, F.J. (1982). Nuclear terminal deoxynucleotidyl transferase in leukaemic infiltrates of testicular tissue. *Br. J. Cancer*, **45**, 709.

TREJDOSIEWICZ, L.K., SMOLIRA, M.A., HODGES, G.M., GOODMAN, S.L. & LIVINGSTON, D.C. (1982). Cell surface distribution of fibronectin in cultures of fibroblasts and bladder derived epithelium: SEM-immunogold localization compared to immunoperoxidase and immunofluorescence. *J. Cell Physiol.*, **123**, 227.

WEISS, L. (1981). Haemopoiesis in mammalian bone marrow. In: *Microenvironments in Haemopoyetic and Lymphoid Differentiation*. (Eds. Porter & Whelan), London: Pitman Med., p. 5.

WESTEN, H. & BAINTON, D.F. (1979). Association of alkaline phosphatase positive reticulum cells in bone marrow with granulocytic precursors. *J. Exp. Med.*, **150**, 919.

WOOD, G.S. & WARNKE, R.A. (1982). The immunologic phenotyping of bone marrow biopsies and aspirates: frozen section techniques. *Blood*, **59**, 913.